# *In Vitro* Engineering of High Modulus Cartilage-Like Constructs

**DOI:** 10.1089/ten.tec.2015.0351

**Published:** 2016-03-09

**Authors:** Scott Finlay, Bahaa B. Seedhom, Duane O. Carey, Andy J. Bulpitt, Darren E. Treanor, Jennifer Kirkham

**Affiliations:** ^1^ Division of Oral Biology, School of Dentistry, University of Leeds, Leeds, United Kingdom.; ^2^ School of Computing, University of Leeds, Leeds, United Kingdom.; ^3^ Department of Pathology, Leeds Institute of Cancer and Pathology, University of Leeds, Leeds, United Kingdom.; ^4^ Leeds Teaching Hospitals NHS Trust, Leeds, United Kingdom.

## Abstract

To date, the outcomes of cartilage repair have been inconsistent and have frequently yielded mechanically inferior fibrocartilage, thereby increasing the chances of damage recurrence. Implantation of constructs with biochemical composition and mechanical properties comparable to natural cartilage could be advantageous for long-term repair. This study attempted to create such constructs, *in vitro*, using tissue engineering principles. Bovine synoviocytes were seeded on nonwoven polyethylene terephthalate fiber scaffolds and cultured in chondrogenic medium for 4 weeks, after which uniaxial compressive loading was applied using an in-house bioreactor for 1 h per day, at a frequency of 1 Hz, for a further 84 days. The initial loading conditions, determined from the mechanical properties of the immature constructs after 4 weeks in chondrogenic culture, were strains ranging between 13% and 23%. After 56 days (sustained at 84 days) of loading, the constructs were stained homogenously with Alcian blue and for type-II collagen. Dynamic compressive moduli were comparable to the high end values for native cartilage and proportional to Alcian blue staining intensity. We suggest that these high moduli values were attributable to the bioreactor setup, which caused the loading regime to change as the constructs developed, that is, the applied stress and strain increased with construct thickness and stiffness, providing continued sufficient cell stimulation as further matrix was deposited. Constructs containing cartilage-like matrix with response to load similar to that of native cartilage could produce long-term effective cartilage repair when implanted.

## Introduction

Cartilage damage can eventually lead to osteoarthritis, causing pain and reduced joint mobility, seriously compromising the affected individual's quality of life.^1^ It is well recognized that cartilage has a poor capacity for spontaneous self-repair, which is, in part, because of its low cellularity and the lack of vascular and lymphatic systems necessary for efficient healing. In addition, any neotissue that is deposited is likely to be destroyed by the stresses acting within joints during daily activities because it is mechanically weak.^2,3^ Intervention is required to maintain quality of life. However, so far, surgical treatments for articular cartilage defects have not been consistently effective in preventing the recurrence of damage.^1,4^

One potential strategy for repairing cartilage is the implantation of cartilage constructs, produced *in vitro,* that have matrix composition and mechanical properties similar to those of the surrounding native cartilage. The principles of tissue engineering would be followed in creating such cartilage constructs, where appropriate mechanical stimulation would be applied onto cells seeded onto a suitable 3D scaffold to promote a chondrocyte-like phenotype and matrix. It is well documented that biochemical stimulation is a prerequisite for successful differentiation/redifferentiation of cells and desired tissue deposition.^5^ Mechanical stimulation is also a highly influential factor in the formation of tissue, especially musculoskeletal tissue.^6^ In order for mechanical loading to elicit a desired mechanotransductive effect on a cartilage-like construct, the following criteria need to be met:

(1) The scaffold should be sufficiently compliant and populated by viable cells with a chondrocyte-like phenotype^7–10^ and surrounded by a cartilage-like matrix such that when loaded, the scaffold will deform and the applied force will be transmitted through the matrix to the cells, and transduced into the cells through appropriate integrins.^11–13^
(2) The values of compressive strain applied onto the constructs should be within the physiological range, which maintain the native tissue's functional properties and are, therefore, likely to induce the desired anabolic effects.^14,15^ *In vitro* cyclic compressive loading regimes shown to elicit beneficial chondroinductive effects on tissue-engineered constructs have applied cyclic strains between 5% and 15% at frequencies of, or near, 1 Hz.^9,16–25^

However, the application of compressive loading to engineered constructs *in vitro* has had limited success in creating mechanically functional constructs that are suitable to implant. This could be because these constructs had moduli that were significantly lower than those of the surrounding native cartilage, they would deform by a greater amount postimplantation, generating excessive shear at the construct–tissue interface impeding integration. In addition, such constructs may be unable to withstand the combination of high compressive and shear stresses arising in the joint. If constructs with mechanical properties comparable to those of the surrounding native cartilage were to be implanted, they are more likely to survive the rigors of the mechanical environment within the joint, integrate with the surrounding native cartilage, and produce long-term repair.^26^

In previous studies, the values of strain (mechanical stimulus) applied onto constructs have been apparently justified solely by their falling within physiological values.^9,20–22^ However, immature constructs have different matrix composition (e.g., much looser structure) and consequently much lower moduli than those of native cartilage. Hence, to produce adequate mechanical stimulus to the cells resident within the constructs, it is likely that applied strain will be greater for these immature constructs than for native cartilage. We hypothesized that determining the response to load of (immature) precultured constructs would help determine a strain that will provide effective mechanical stimulation to the residing cells within the construct. This would then lead to the formation of cartilage-like constructs with biochemical composition and high-compressive moduli similar to those of native cartilage, and so be mechanically functional.

The objectives of this study were, therefore, to (1) determine appropriate parameters for mechanical stimulation, namely the range of strains to be applied on the constructs; (2) develop a method for implementing the desired mechanical stimulation; (3) compare the compressive properties of the constructs that were subjected to this mechanical stimulus with those of nonloaded controls and native cartilage; and (4) potentially identify any histological characteristics of the constructs that might be associated with their compressive properties.

## Materials and Methods

**FIG. 1.**
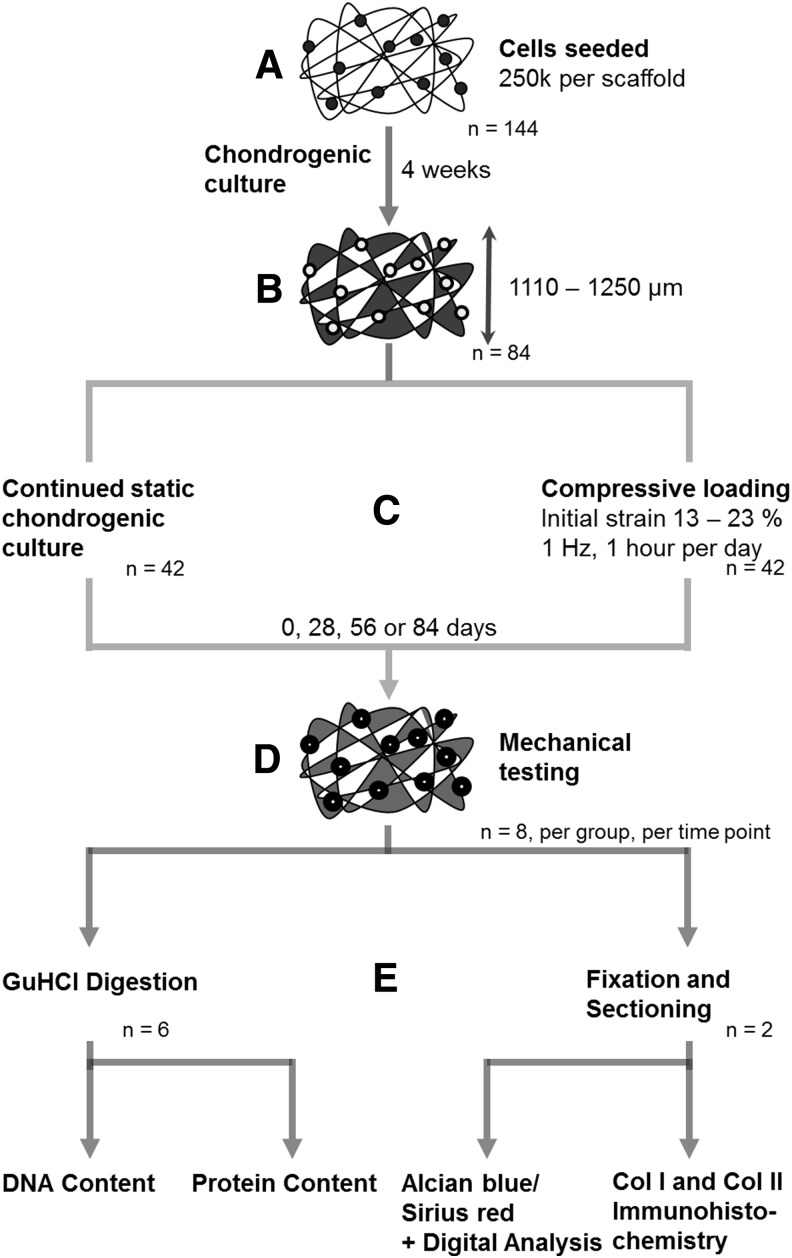
The experimental stages in creating cartilage-like constructs and their analyses. **(A)** Nonwoven polyethylene terephthalate scaffolds were each seeded with 250,000 synoviocytes and subjected to chondrogenic medium for 4 weeks. **(B)** Constructs of thicknesses between 1110 and 1250 μm were then selected for continued culture to produce desired strain upon compressive loading. **(C)** Constructs were either cultured under static chondrogenic conditions or additionally subjected to compressive loading at an initial strain between 13% and 23%, at 1 Hz for 1 h per day, for 0, 28, 56, or 84 days. **(D)** At each time point, eight constructs from each experimental group were mechanically tested. **(E)** Six of these constructs were digested with GuHCl and DNA and protein content was measured. Two of these constructs were fixed and sectioned, and stained with Alcian *blue*/Sirius *red* and antibodies were raised against type I and type II collagen. Alcian *blue*/Sirius *red*-stained sections were digitally analyzed.

A flow diagram depicting the overall experimental and analytical stages is shown in Figure 1.

### Cell acquisition and monolayer expansion

Bovine synoviocytes were obtained from the synovia of 6-month-old bovine metatarsophalangeal joints, using a method similar to that described by De Bari *et al.* 2001.^27^ Synoviocytes were selected because of their strong chondrogenic potential^28–30^ and, should such a strategy be translated, extraction of a biopsy should not cause long-term damage. Each synovial sample was cut into small pieces and digested in 0.25% (w/v) collagenase 1A (Sigma Aldrich, Gillingham, United Kingdom; C9891) within Dulbecco's modified Eagle's medium/Ham's F12 medium (DMEM/F12) (Invitrogen, Paisley, United Kingdom; 21041) at 37°C for 3 h. The digest was centrifuged at 100 *g* for 10 min and the resulting pellet was resuspended in DMEM/F12 and seeded at 5000 cells/cm^2^ into tissue culture flasks after filtering through a 70 μm nylon filter. Synoviocytes were cultured in DMEM/F12 supplemented with 10% fetal bovine serum (FBS), 1% 100× antibiotic (AB) (Sigma Aldrich; P0781), and 2 mM l-glutamine at 37°C and 5% CO_2_. When approaching 90% confluency, cells were passaged through trypsinization and reseeded at 5000 cells/cm^2^. Cells between passages 1 and 3 were used for experimentation.

### Scaffold cell seeding and static chondrogenic culture

Polyethylene terephthalate (PET) scaffolds were used for experimentation. These were comprised of nonwoven 20 μm diameter filaments that had been plasma treated (Xiros Ltd., Leeds, United Kingdom).^31^ Scaffolds had an overall porosity of 90.2% by volume and were in the form of disks of 5 mm diameter and 0.9 mm thickness. A 250,000 cells/mL suspension was prepared and 1 mL of cell suspension was combined with one PET scaffold and placed within a 2 mL capacity polypropylene tube. Each tube was then inserted into an in-house built, dynamic cell-seeding apparatus in which these tubes were rotated at a rate of approximately 0.25 Hz. The dynamic cell-seeding apparatus was placed into an incubator at 37°C for 24 h to allow cells to attach to the PET scaffold. Each scaffold was then transferred into a single well of a 24-well tissue culture plate and submerged within 1 mL of chondrogenic medium, which consisted of DMEM/F12 supplemented with 10 ng/mL TGF-β3 (Invitrogen; PHG9305), 10^–7^ M dexamethasone, 1 × Insulin-Transferrin-Selenium (Invitrogen; 51300044), 50 μg/mL l-ascorbic acid-2-phosphate sesquimagnesium salt hydrate (Sigma Aldrich; A8960), 1 × AB, and 2 mM l-glutamine. The plates were placed in an incubator at 37°C. Cultures remained under these conditions for a total of 4 weeks. The medium was replaced every 3–4 days.

### Response of immature constructs to load

**FIG. 2.**
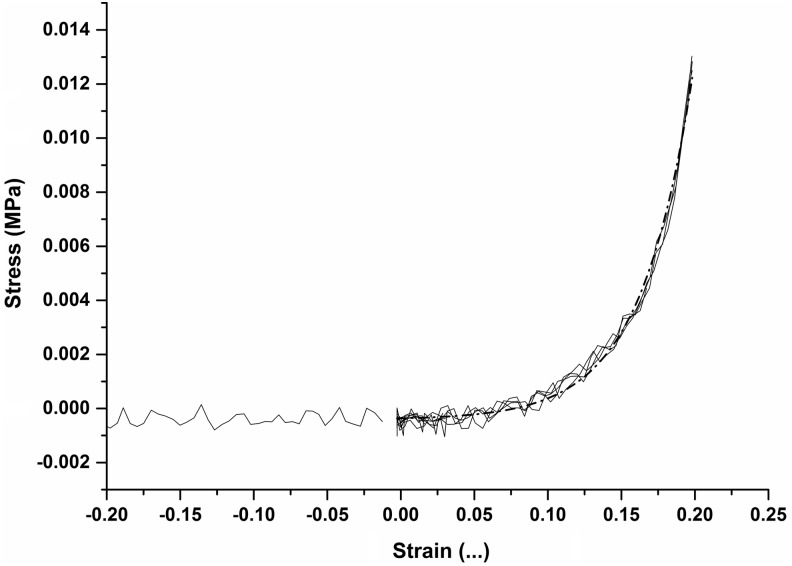
Typical engineering stress versus strain curve acquired from dynamic mechanical testing of constructs after 4 weeks in chondrogenic culture, showing zero load reading and loading curves from final 4 cycles only (of a total of 10 cycles). Exponential fit (*dotted line*) made between 0.0 and 0.2 strain, at which tangent instantaneous moduli were determined.

To identify a suitable loading regime to apply onto the immature constructs, their mechanical response to load needed to be ascertained. After 4 weeks in chondrogenic culture, 13 constructs were each subjected to 20% cyclic strain, at a frequency of 1 Hz, within a dynamic universal testing machine (ElectroPuls E3000, Instron), and the corresponding load was recorded. A representative example of the data is shown in Figure 2. The data showed that the loads recorded at strains of less than 10% were almost negligible and consequently little stress was being transmitted to the matrix and its resident cells. However, at strains greater than approximately 13%, the measured load increased exponentially, indicating the potential for stress to be transmitted to the matrix and residing cells, which was hoped would be adequate to elicit the desired mechanotransductive effects. This strain value of 13% was, therefore, selected as the minimum strain to be applied to the immature constructs. An upper limit of 30% strain was chosen. This value tended toward the higher limit of strains observed in native cartilage, but we assumed that the loose matrix populating the construct was not likely to transmit damaging forces to the cells.

**FIG. 3.**
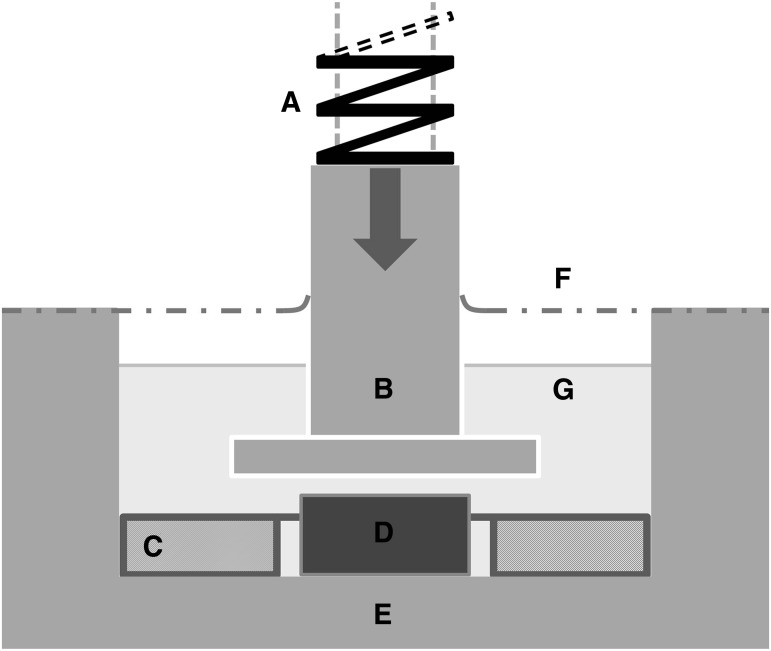
Schematic representation of the bioreactor used to apply compressive loading to the developing constructs (single well shown, actual 12 wells in total). **(A)** precompressed spring, **(B)** stainless steel plunger, **(C)** silicone ring, **(D)** construct, **(E)** stainless steel well, **(F)** polyurethane film, **(G)** chondrogenic culture medium. The bioreactor is driven by a 12 volt DC motor connected to an assembly comprising a gear box, timing belt, and pulley system, which causes the plungers to reciprocate at a rate of 1 Hz. The force delivered by the plunger comprises the weight of the plunger assembly and the load exerted by a precompressed spring. A flexible polyurethane film seals the rim of well to prevent the culture medium from contamination. The plunger penetrates the film, which also seals the exit of the plunger, but because it is flexible the film does not constrain the plunger's vertical movement. The film allows gaseous exchange between its two sides and maintains sterility of the culture wells.

Application of the selected strain range was achieved using an in-house built compressive bioreactor. The bioreactor was driven by a 12 volt DC motor connected to an assembly comprising a gear box, timing belt, and pulley system, which caused 12 individual plungers to reciprocate vertically at a frequency of 1 Hz. The bioreactor was force controlled. The force delivered by each plunger (which was 7 mm in diameter) comprised the weight of the plunger assembly and the load exerted by a precompressed spring. Each construct was cultured in a separate well of a custom chamber, comprising six wells, made of (316) stainless steel, as shown in Figure 3. A flexible polyurethane film (OpSite Flexigrid, Smith & Nephew, Hull, United Kingdom; 4631) sealed the rim of each well. The plunger penetrated the film, which also sealed the exit of the plunger but did not constrain the plunger's vertical movement. The film allowed for gaseous exchange while maintaining sterility of the culture wells.

The following setup was adapted to apply mechanical loading in a quasistrain controlled modality. The plunger displacement was limited by surrounding each construct with a silicone ring of 1 mm in thickness and compressive modulus of 2.64 MPa (produced from Sylgard 184 elastomer, VWR International; Lutterworth, United Kingdom; 634165S), as shown in Figure 3. The silicone ring had substantially greater stiffness than the constructs after their 4-week incubation in chondrogenic culture. The effect of the constructs on the plunger displacement was assumed to be negligible when the bioreactor applied a relatively high force of 5 N. Hence, the initial plunger displacement could be determined from (1) the force applied by the bioreactor plunger, (2) the stiffness of the silicone ring, and (3) the contact surface areas of the plunger with the silicone ring and construct. Construct strain was, therefore, dependent on plunger displacement and construct thickness.

To achieve the desired strain, constructs of thicknesses between 1110 and 1250 μm were selected. Thickness measurement at this stage of experimentation was achieved using sterile Vernier callipers having an accuracy of 10 μm. This corresponded to a strain between 13% and 23%. Thirty five per cent of the constructs was discarded, because their thicknesses did not fall within the desired range after 4 weeks in static chondrogenic culture.

Constructs were loaded for 1 h per day, 5 days per week for 28, 56, or 84 days. Experimental controls (nonloaded constructs) were returned to culture within tissue culture plastic well plates. Chondrogenic medium was exchanged every 3–4 days.

### GuHCl digestion, DNA and protein quantification

Before DNA and protein quantification, the constructs' contents were solubilized. Constructs were removed from the culture, dipped into phosphate-buffered saline (PBS) (to remove excess medium), and then placed into 1 mL of 4 M GuHCl with 50 mM Tris (pH 7.5) and vortexed at 4°C for 30 min.^32^ The resulting solution was stored frozen at −20°C until analyzed (*n* = 6 for each time point).

DNA quantification was accomplished using the Quant-iT™ PicoGreen^®^ assay (Invitrogen; P7581); a 30 μL aliquot of GuHCl digested construct was added to 900 μL of TE buffer (10 mM Tris-HCl and 1 mM EDTA, at pH 7.5) and vortexed. Fifty microliters of this was then combined with 50 μL PicoGreen working solution (10 μL PicoGreen concentrate, 1990 μL TE buffer) and placed in a 200 μL capacity PCR tube. Fluorescence was read at 520 nm (excited at 480 nm).

Protein content was determined by Bradford assay; a 5 μL aliquot of GuHCl digested construct was added to 250 μL of Quick Start™ Bradford Dye Reagent (Bio-Rad Laboratories, Hemel Hempstead, United Kingdom; 500–0205) within a well of a 96-well plate. The solution was pipette mixed and incubated at room temperature for 5 min. Absorbance was read at 595 nm.

### Mechanical testing

The compressive moduli of native cartilage and constructs at each time point (days 0, 28, 56, and 84) were measured. Constructs were transferred to PBS and their mechanical properties were determined within 30 min. Constructs were dynamically tested using an Instron ElectroPuls™ E3000 materials testing machine. The thickness of each construct was first determined as follows: the construct was placed between two metallic platens and suspended in a column of PBS. The top platen was lowered at a rate of 50 μm/s and stopped once a tare load of −0.02 N was measured. The construct thickness was read as the distance between the two facing surfaces of the platens. The construct was then subjected to 10 cycles of 20% cyclic strain at 1 Hz, and both the load and resulting compression of the construct were simultaneously recorded.

A dynamic testing method was chosen because it simulates functional loading and, therefore, a functional response of the constructs.^33^ In addition, in studies that have compared mechanical testing data of constructs with those of native cartilage, although equilibrium mechanical properties have been comparable to those of native articular cartilage, the dynamic properties have been substandard.^21,34^ Construct diameter was estimated by matching the constructs to circles of known diameter, increasing in increments of 100 μm.

The 4 last cycles (out of 10) were chosen for mechanical testing as it was shown that a steady state of the recorded loading profile had been achieved. A stress–strain curve was plotted from the data of the four cycles, to which a best-fit exponential curve was fitted. The gradient of this curve at strain values of 10%, 12%, 15%, and 18% was calculated by differentiating the exponential best-fit curve. The resulting values were equivalent to the tangent instantaneous moduli of the construct at these strains (*n* = 6–8 at each time point).

Bovine articular cartilage specimens were tested in the same manner as the constructs. The test specimens were obtained as described by Fortin *et al.* 2000^35^: osteochondral plugs, 7 mm in diameter, were harvested (with a rotating reamer) from the flattest surfaces of the trochlea of a bovine knee joint of a 6-month-old animal. The subchondral bone was removed from the osteochondral plugs with a precision rotary saw to leave as close to full depth cartilage as possible (*n* = 8).

### Fixation, embedding, and sectioning of constructs

Constructs were removed from the culture, washed with PBS to remove excess medium, and fixed in 10% neutral buffered formalin for at least 30 min (*n* = 2 at each time point). Fixed constructs were then washed in PBS. Embedding and sectioning of these constructs were carried out by Covance Laboratories Ltd. (Harrogate, United Kingdom). After fixing but before embedding, a 3 mm wide central region was cut from the 5 mm diameter construct disks, using a scalpel. The cut region was then embedded in paraffin wax within an enclosed tissue processor, with the cut face positioned parallel to the resulting block to create longitudinal sections with respect to the disk face. In brief, samples were then dehydrated in a series of increasing concentrations of ethanol, followed by multiple treatments of xylene to remove the ethanol. Finally, melted paraffin wax was infiltrated into the sample with the aid of vacuum. Once the wax had cooled and solidified, the samples were sectioned with a rotary microtome to a thickness of 5 μm and mounted onto slides.

### Histological staining and light microscopy of sectioned constructs

#### Alcian blue/Sirius red staining methodology

Construct sections were taken to water and then stained with Weigert's hematoxylin (10 min). Excess stain was removed by a water wash (10 min), followed by a brief dip in 1% HCl (in methanol). Sections were stained with 0.5% Alcian blue 8GX (in 1% acetic acid in water) for 10 min. Excess stain was removed by a water wash (1 min). Sections were further stained with 0.3% Sirius red (in saturated picric acid) for 45 min. Excess stain was removed by a water wash (1 min). Sections were then dehydrated, cleared, and mounted in p-xylene bis-pyridinium bromide (DPX).

#### Immunohistochemistry of construct sections

Construct sections were taken to water and then immersed in 2% H_2_O_2_ (20 min). After washing in PBS, antigen was retrieved by submerging the sections in 0.1% chymotrypsin (Sigma Aldrich; C4129) at 37°C, pH 7.8 (within 0.1% CaCl_2_) (30 min), followed by another wash in PBS. Sections were then blocked with normal goat serum (20 min) to reduce nonspecific binding, washed with PBS, then incubated with a primary antibody overnight at 4°C. Primary antibodies used were raised against antigens for collagen type I (Abcam, Cambridge, United Kingdom; monoclonal ab6308) and collagen type II (Calbiochem, Nottingham, United Kingdom; monoclonal II-4C11) and were used at dilutions of 1 in 300 and 1 in 1500, respectively. Sections were then washed with PBS and incubated with secondary antibodies as per the EnVision™ kit's instructions (Dako, Ely, United Kingdom; K5007) (30 min). After incubation, sections were washed again with PBS and exposed to the chromogenic substrate (10 min). Developed sections were washed with water, counterstained within Harris's hematoxylin (3 min), water washed, and immersed in Scott's tap water (3 min) before a final water wash. Sections were then cleared by two washes in xylene (2 min each). Finally, the sections were mounted in DPX and observed by standard light microscopy.

**FIG. 4.**
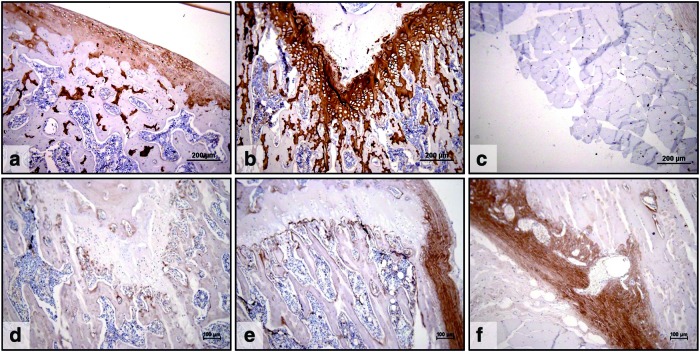
Histological appearance of control sections from decalcified Wistar rat femurs. Sections stained with antibodies to collagen type II **(a, b, c)** and antibodies to collagen type I **(d, e, f)**. **(a)** strong positive collagen type II staining of the articular cartilage, **(b)** strong positive collagen type II staining of the growth plate, **(c)** no collagen type II staining was seen in muscle, **(d)** no collagen type I staining was seen in growth plate, but positive type I staining was evident in cancellous bone, **(e)** no collagen type I staining was observed in growth plate, but positive staining was evident in cancellous bone and strong positive staining was present in cortical bone, **(f)** strong positive collagen type I staining was seen in cortical bone. Scale bar **a**, **b,** and **c** = 200 μm. Scale bar **d**, **e,** and **f** = 100 μm. Color images available online at www.liebertpub.com/tec

**FIG. 5.**
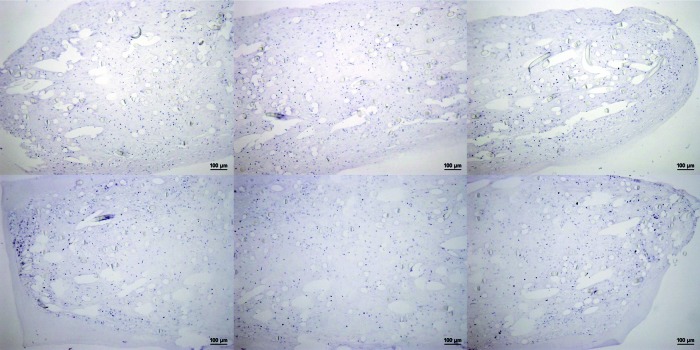
Negative controls for immunohistochemistry of constructs. Histological appearance of sections from two constructs at day 84 of mechanical loading stained without the inclusion of either collagen type I or collagen type II primary antibody. Three images, taken from the midpoint and each end of the construct, are shown in each case, representing almost the entire construct. Scale bar 100 μm. Color images available online at www.liebertpub.com/tec

To ensure that the primary antibodies used were specific for the epitopes being investigated, they were tested against native cartilage, growth plate, and cortical and trabecular bone from mammalian joints (Fig. 4). In addition, to ensure the secondary antibodies and chromogen development methodology did not exhibit false positive staining, sections without primary antibodies were also produced (Fig. 5).

### Image analysis of histochemical staining

Sections that were stained with Alcian blue and Sirius red were analyzed to determine the percentage coverage and mean staining intensity of Alcian blue histochemical stain. An Aperio T2 whole slide imaging device (Aperio, Vista, CA) was used to acquire images of the sectioned engineered constructs using a ×20 objective, resulting in images with a spatial resolution of 0.49 μm.^36^ The Alcian blue percentage coverage and mean intensity of these ultra-high-resolution images were evaluated by custom written image analysis software written in Matlab (Natwick USA) that used color deconvolution^37^ and Otsu's thresholding^38^ method.

Color deconvolution is a methodology that changes the bases of the original images' red, blue, and green channels into those that are representative of user-defined reference stains. To automatically generate reference stains, Macenko *et al.*'s singular value decomposition method was used.^39^

Color deconvolution uses a simple least squares fit and so the calculated staining coefficients for all image's pixels needed to be differentiated into actual and nonstaining contributions. This was achieved by Otsu's method,^38^ which finds a threshold on an image's staining intensity histogram by maximizing the variation between the hypothesized staining contributions, that is, actual and nonstained regions.

The threshold was derived from an entire whole slide image so that inconsistent segmentation was avoided. To negate issues relating to noise and histological artifacts outside the area of interest, manual demarcations were made around the periphery of the sections using Aperio Image Scope software (version 10) to restrict image analysis to the chosen areas. The positive pixels found within the delineated region were used to compute percentage coverage and mean staining intensity metrics for each image. These image analysis values were then plotted against the base 10 logarithm of each sample's instantaneous compressive modulus at 18% strain.

### Statistical analysis

Data are presented as mean ± standard deviation (SD). Statistical significance between two datasets was determined by Mann–Whitney test. Statistical significance is presented by superscript a, b, or c to denote when *p* < 0.05, *p* < 0.01, and *p* < 0.001, respectively.

## Results

### Protein and DNA content of constructs

**FIG. 6.**
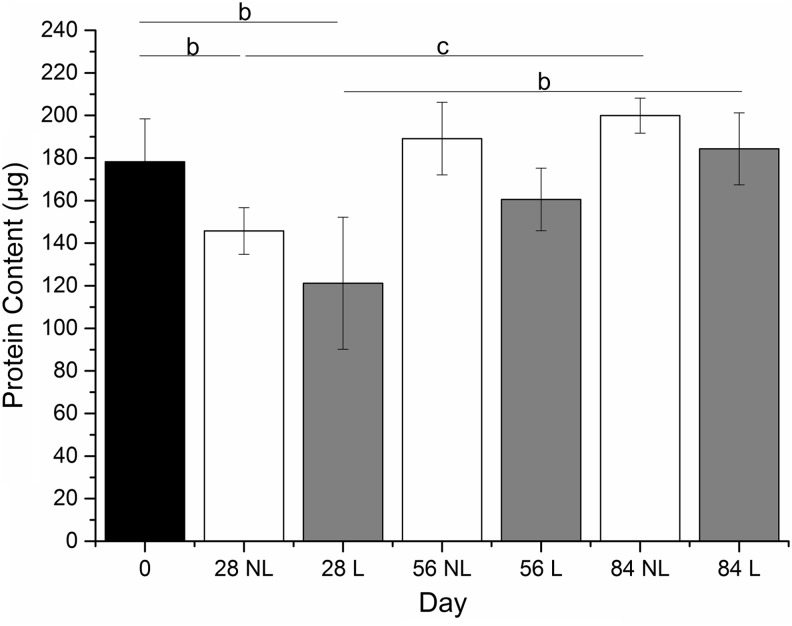
Protein content of synoviocyte/PET constructs precultured in chondrogenic medium for 4 weeks (day 0) and then subjected to a mechanical loading regime for 28, 56, or 84 days. No significant differences were seen between loaded and nonloaded constructs at each individual time point. Significant decrease in protein content was observed from days 0 to 28 and significant increases from days 28 to 84 in both loaded and nonloaded constructs. Results are expressed as mean ± SD (*n* = 6). ^c^*p* < 0.01, ^b^*p* < 0.001. PET, polyethylene terephthalate; NL, nonloaded; L, loaded.

From days 0 to 28, there was a significant decrease in the protein content for both loaded and nonloaded constructs; then throughout the remaining culture period (of 84 days), the protein content gradually increased in both experimental groups (Fig. 6). There was no statistical difference between loaded and nonloaded constructs at each time point.

**FIG. 7.**
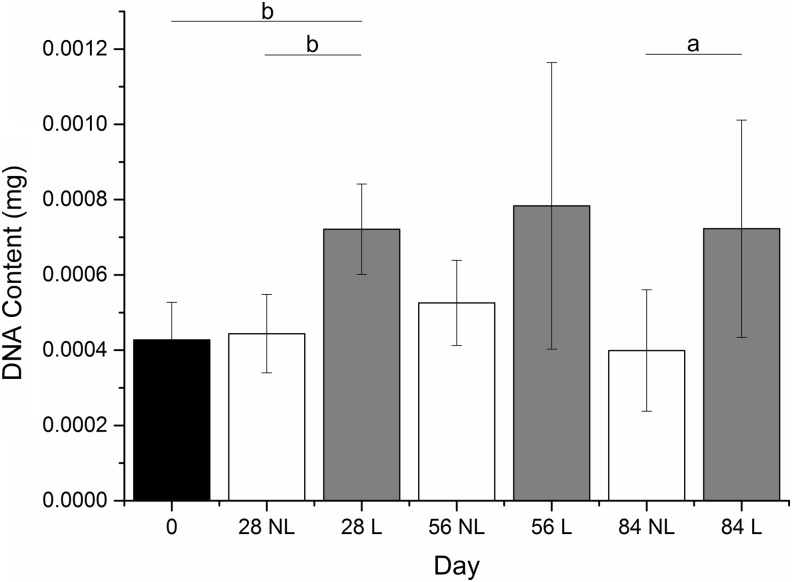
DNA content of synoviocyte/PET constructs precultured in chondrogenic medium for 4 weeks (day 0) and then subjected to a mechanical loading regime for 28, 56, or 84 days. Mechanical loading was associated with an increase in cell numbers within the first 28 days in comparison to nonloaded controls, this significance was not present at day 56, but was present at day 84. Cellular content, as judged by DNA content, remained consistent throughout culture. Results are expressed as mean ± SD (*n* = 6). ^a^*p* < 0.05, ^b^*p* < 0.01.

The cellular content of the nonloaded constructs (as judged by DNA content) did not change significantly throughout the entire 84 days of culture (Fig. 7). Loaded constructs, however, had an apparent surge in cell proliferation within the first 28 days and continued to have high-cellular content for the remaining culture period (to 84 days) (Fig. 7).

### Mechanical testing of constructs

#### Construct thickness, construct diameter, and estimated applied strain onto loaded constructs

**FIG. 8.**
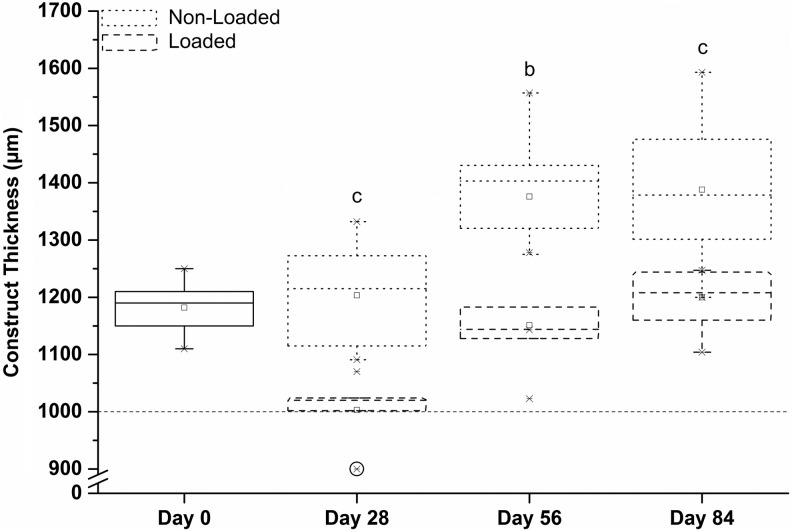
Thickness of the developing synoviocyte/PET constructs at different time points during culture with and without the presence of mechanical loading. *Dotted line* indicates the thickness of the silicone ring that surrounded the construct during mechanical loading. *Circle* highlights a sample that atrophied and was thinner than the silicone ring and henceforth was not subjected to strain. The thickness of nonloaded constructs increased until day 56 and remained at this thickness at day 84. Construct thickness reduced from days 0 to 28, followed by an increase for the remaining culture period. Nonloaded constructs were thicker than loaded constructs at all time points ^b^*p* < 0.01, ^c^*p* < 0.001.

The thickness of nonloaded constructs increased significantly (*p* < 0.001) until day 56 and remained at this thickness at day 84. The thickness of the loaded constructs significantly reduced from days 0 to 28, after which the thickness increased for the remaining culture period. The rate of increase in thickness was greatest from days 28 to 56. Nonloaded constructs were significantly thicker than loaded constructs at all time points (Fig. 8).

**FIG. 9.**
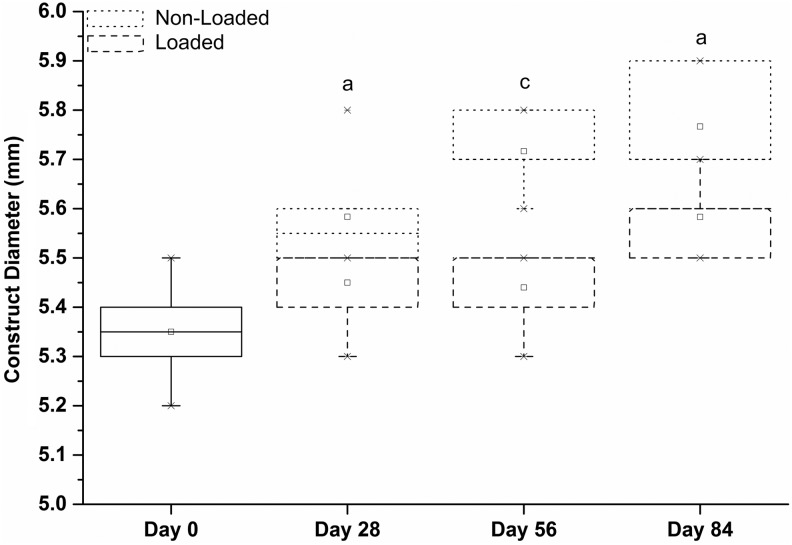
Diameter of the developing synoviocyte/PET constructs at different time points during culture with and without the presence of mechanical loading. The diameter of nonloaded constructs increased at each time point. The loaded construct diameter did not statistically increase in diameter until day 84 of mechanical loading. Nonloaded constructs had a larger diameter than loaded constructs at all time points. ^a^*p* < 0.05, ^c^*p* < 0.001.

The diameter of nonloaded constructs increased at each time point, although this was not significant between days 56 and 84. However, the rate of enlargement decreased with time. The loaded constructs did not have a statistically significant increase in diameter until day 84 of mechanical loading. Nonloaded constructs had significantly larger diameter than loaded constructs at all time points (Fig. 9).

**FIG. 10.**
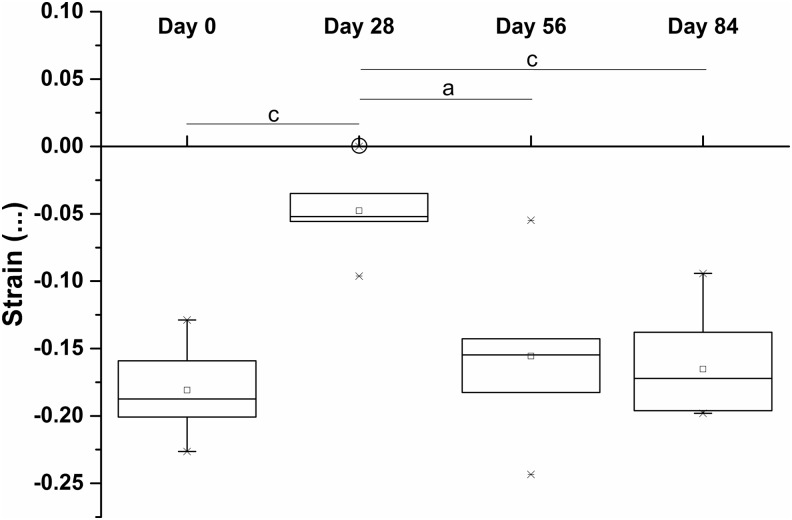
Estimated strain applied to the developing synoviocyte/PET constructs during mechanical loading, based on mechanical testing data. *Circle* highlights a sample that atrophied and was thinner than the silicone ring, henceforth was not subjected to strain. Estimated strain decreased from days 0 to 28, followed by an increase from days 28 to 56. ^a^*p* < 0.05, ^c^*p* < 0.001.

The estimated applied strain at each time point was determined using the thickness and moduli of the constructs and thickness and modulus of the silicone ring. The applied strain followed a trend similar to that of construct thickness. The applied compressive strain decreased significantly from days 0 to 28. From days 28 to 56 it increased significantly. There was no significant increase from days 56 to 84 (Fig. 10).

#### Compressive moduli

The compressive moduli values (expressed as tangents to stress–strain curves), which were measured at different strains (on the stress–strain plots) and at time points of the study, are compared in Tables 1 and 2.

**Table 1.** Tangential Compressive Moduli of Nonloaded Synoviocyte/Polyethylene Terephthalate Constructs After Preculture in Chondrogenic Medium for 4 Weeks (Day 0) for 28, 56, or 84 Days and Native Bovine Trochlear Cartilage at Strains of 10%, 12%, 15%, and 18%

	*Modulus at 10% strain (± SD) (MPa) [range]*	*Modulus at 12% strain (± SD) (MPa) [range]*	*Modulus at 15% strain (± SD) (MPa) [range]*	*Modulus at 18% strain (± SD) (MPa) [range]*
Day 0	0.025 (± 0.006) [0.017–0.033]	0.037 (± 0.015) [0.021–0.060]	0.067 (± 0.043) [0.025–0.146]	0.128 (± 0.115) [0.031–0.353]
Day 28	0.011 (± 0.003) [0.005–0.014]	0.028 (± 0.008) [0.015–0.037]	0.114 (± 0.040) [0.074–0.188]	0.484 (± 0.229)^b^ [0.207–0.936]
Day 56	0.008 (± 0.006) [0.002–0.017]	0.025 (± 0.015) [0.007–0.045]	0.152 (± 0.062) [0.048–0.225]	0.968 (± 0.342)^a^ [0.326–1.311]
Day 84	0.023 (± 0.019) [0.006–0.063]	0.076 (± 0.058)^a^ [0.024–0.197]	0.445 (± 0.311)^a^ [0.260–1.091]	2.680 (± 1.700)^a^ [1.385–8.029]
Native	0.182 (± 0.267) [0.015–0.677]	0.447 (± 0.624) [0.036–1.582]	1.803 (± 2.228) [0.139–5.649]	7.849 (± 8.531) [0.532–20.167]

Statistical significance of moduli values was in comparison with moduli values from previous time point (*n* = 6–8).

^a^ *p* < 0.05.

^b^ *p* < 0.01.

**Table 2.** Tangential Compressive Moduli of Loaded Synoviocyte/Polyethylene Terephthalate Constructs Under Cyclic Strain After Preculture in Chondrogenic Medium for 4 Weeks (Day 0) for 28, 56, or 84 Days and Native Bovine Trochlear Cartilage at Strains of 10%, 12%, 15%, and 18%

	*Modulus at 10% strain (± SD) (MPa) [range]*	*Modulus at 12% strain (± SD) (MPa) [range]*	*Modulus at 15% strain (± SD) (MPa) [range]*	*Modulus at 18% strain (± SD) (MPa) [range]*
Day 0	0.025 (± 0.006) [0.017–0.033]	0.037 (± 0.015) [0.021–0.060]	0.067 (± 0.043) [0.025–0.146]	0.128 (± 0.115) [0.031–0.353]
Day 28	0.047 (± 0.046) [0.020–0.127]	0.116 (± 0.100) [0.044–0.230]	0.458 (± 0.322) [0.210–0.999]	1.840 (± 1.030)^a^ [1.060–4.094]
Day 56	0.129 (± 0.070) [0.074–0.223]	0.398 (± 0.185) [0.255–0.630]	2.180 (± 0.829)^a^ [1.336–2.996]	12.09 (± 4.160)^b^ [7.311–16.633]
Day 84	0.211 (± 0.175) [0.066–0.590]	0.587 (± 0.404) [0.198–1.443]	2.800 (± 1.400)^a^ [1.031–5.513]	15.22 (± 4.210)^b^ [8.923–18.425]
Native	0.182 (± 0.267) [0.015–0.677]	0.447 (± 0.624) [0.036–1.582]	1.803 (± 2.228) [0.139–5.649]	7.849 (± 8.531) [0.532–20.167]

Statistical significance of moduli values was in comparison with moduli values from previous time point (*n* = 6–8).

^a^ *p* < 0.05.

^b^ *p* < 0.01.

**FIG. 11.**
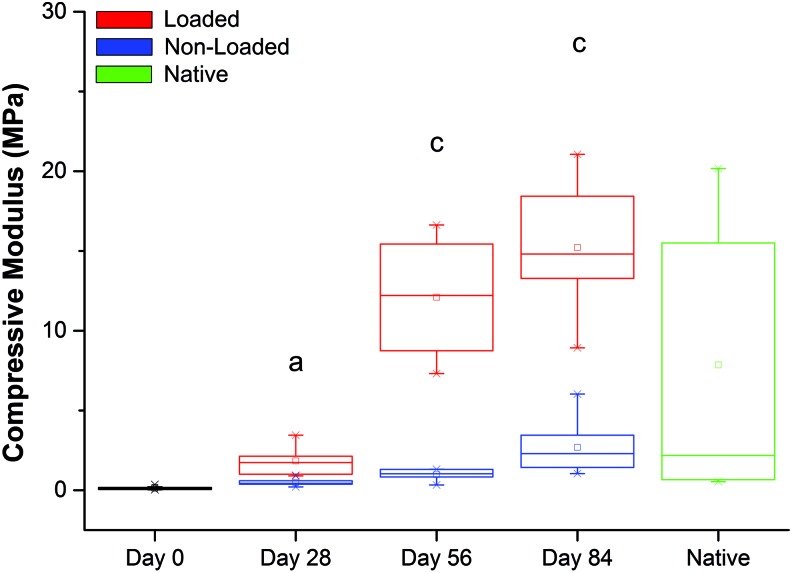
Compressive moduli measured at 18% strain of native bovine trochlear cartilage (*green*) and synoviocyte/PET constructs under 1 Hz 20% cyclic strain after preculture in chondrogenic medium for 4 weeks (day 0) and then subjected to a mechanical loading regime for 28, 56, or 84 days. Moduli of loaded constructs were greater than those of nonloaded constructs throughout, substantially so at days 56 and 84 (*n* = 6–8). ^a^*p* < 0.01, ^c^*p* < 0.001. Color images available online at www.liebertpub.com/tec

Moduli of both the loaded and nonloaded constructs tended to increase as the experiment progressed in time, but not in a consistent manner. Furthermore, the loaded constructs had significantly higher moduli throughout the experiment than the nonloaded constructs (Fig. 11). Also, as a construct was loaded, the higher the strain applied, the higher was the value of the tangent modulus observed.

At 18% strain, the moduli of the nonloaded constructs increased steadily at each time point, from a mean of 0.13 MPa at day 0 (i.e., at the end of the 4-week culture period) to an average of 2.7 MPa at day 84 (Table 1). Moduli of loaded constructs increased significantly at days 28 and 56 (mean of 1.8 MPa and 12.1 MPa, respectively) compared with the previous time point. Although the modulus of constructs continued to increase in the period between days 56 (12.1 MPa) and 84 (15.2 MPa), this increase was not statistically significant (*p* = 0.290) (Table 2). Thus the greatest effect of compressive mechanical loading on construct compressive moduli occurred between days 28 and 56. Native bovine articular cartilage from the trochlea of the knee had an average modulus of 7.8 MPa (ranging from 0.5 to 20.2 MPa).

At 10% strain, no change was observed in the modulus of the nonloaded constructs at any time point (Table 1). At 12% strain, a significant difference in modulus was observed between days 56 and 84, although this was not substantial in absolute terms. At 15% strain, a significant difference in the modulus was observed between days 56 and 84. Thus, only at 18% strain was there a clearly observable significant difference in moduli at each time point.

The moduli of *loaded* constructs obtained at 10% strain did not show significant differences at the various time points (Table 2). However, at day 84, in contrast to nonloaded constructs, the moduli were similar to those of native cartilage. At 12% strain, the moduli values continued to be comparable to those of native cartilage at days 56 and 84. At 15% strain, there were significant differences between days 28 and 56, and days 56 and 84. This significance increased at 18% strain, with moduli values comparable to the modulus values at the higher end of the range of native cartilage.

### Histological appearance of constructs

**FIG. 12.**
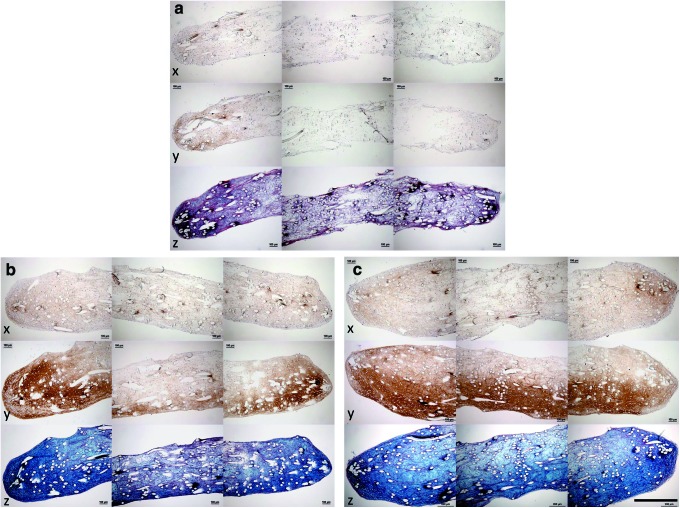
Histological appearance of sections from constructs at **(a)** day 0, **(b)** nonloaded day 28, and **(c)** loaded day 28 of culture with the presence of mechanical loading. Sections stained with (x) antibodies to collagen type I, (y) antibodies to collagen type II, and (z) Alcian *blue*/Sirius *red*. Three images, taken from the midpoint and each end of the construct, are shown in each case, representing almost the entire construct. Scale bar 500 μm. Color images available online at www.liebertpub.com/tec

**FIG. 13.**
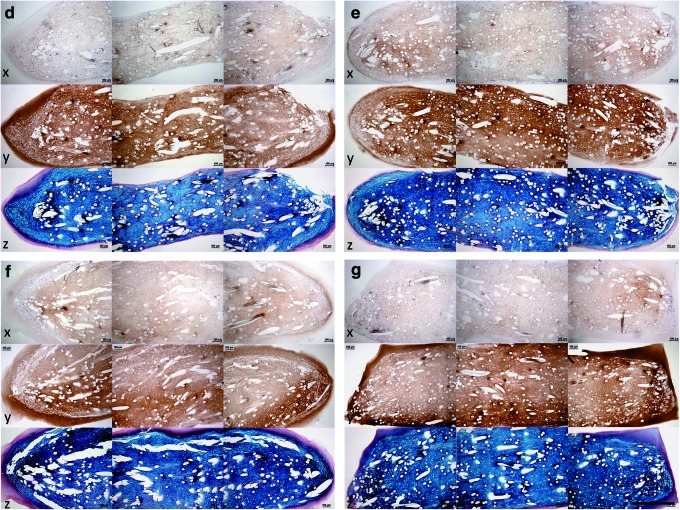
Histological appearance of sections from constructs at **(d)** nonloaded day 56, **(e)** loaded day 56, **(f)** nonloaded day 84, and **(g)** loaded day 84 of culture with the presence of mechanical loading. Sections stained with (x) antibodies to collagen type I, (y) antibodies to collagen type II, and (z) Alcian *blue*/Sirius *red*. Three images, taken from the midpoint and each end of the construct, are shown in each case, representing almost the entire construct. Scale bar 500 μm. Color images available online at www.liebertpub.com/tec

The histological appearance of the constructs in the presence or absence of loading and at different time points in the culture period is shown in Figures 12 and 13. At day 0 of the mechanical loading (after 4 weeks of preculture), low level staining was seen for collagen type I, collagen type II, and Alcian blue (Fig. 12a), primarily localized at the periphery of the constructs' cross-sections.

By day 28, there was a considerable increase in staining for collagen type II and Alcian blue in both loaded and nonloaded constructs (compared with day 0), suggesting the deposition of a cartilage-like matrix. The laying down of cartilage-like matrix appeared to first occur at the edges of the construct and at either the top or bottom face of the construct (Fig. 12b, c). There was an increased amount of collagen type I staining throughout all constructs compared with day 0.

By day 56, there was further increase in staining for collagen type II and Alcian blue in both loaded and nonloaded constructs accompanied by increased construct thickness, in comparison with those at day 28. In addition, at day 56 there were visible differences in histological appearance between loaded and nonloaded constructs. Homogeneity of staining for collagen type II and Alcian blue throughout the construct volume was different according to whether the constructs had been loaded or not. Loaded constructs (Fig. 13e) had greater homogeneity of staining than nonloaded constructs (Fig. 13d). In addition, loaded constructs had a more uniform shape than nonloaded constructs.

At day 84, the histological appearance of the constructs was similar to that seen at day 56, including the differences previously observed between loaded and nonloaded constructs. Nonloaded constructs had a variable cross-sectional shape and nonhomogeneous matrix staining for collagen type I, collagen type II, and Alcian blue (Fig. 13f). Loaded constructs had uniform shape throughout their cross-section and homogeneous matrix staining throughout (Fig. 13g).

### Image analysis of histochemical staining

**FIG. 14.**
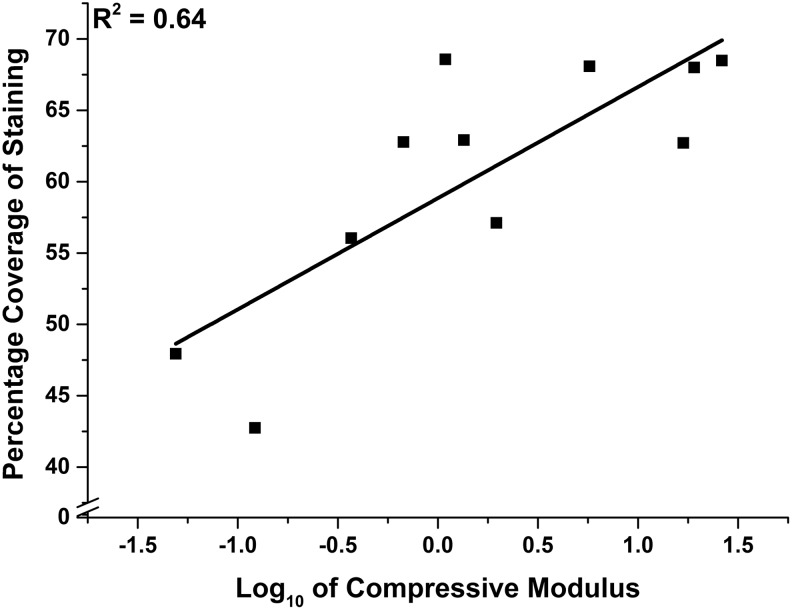
Percentage coverage of Alcian *blue*-stained sections of synoviocyte/PET constructs versus the corresponding base 10 logarithm of instantaneous compressive modulus at 18% strain showing line of best fit (*R*^2^ = 0.64).

**FIG. 15.**
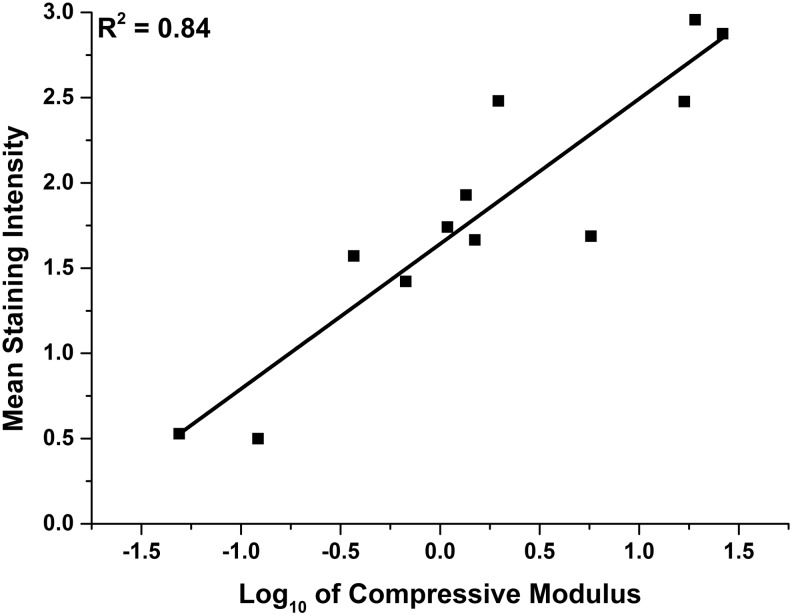
Mean staining intensity of Alcian *blue*-stained sections of synoviocyte/PET constructs versus the corresponding base 10 logarithm of instantaneous compressive modulus at 18% strain showing line of best fit (*R*^2^ = 0.84).

The percentage coverage and mean staining intensity of Alcian blue within both loaded and nonloaded constructs demonstrated a positive linear relationship with the base 10 logarithm of the instantaneous compressive modulus, with *R*^2^ values of 0.64 and 0.84, respectively (Figs. 14 and 15). Mean staining intensity correlated more closely with compressive modulus than with percentage coverage of the stain.

## Discussion

The ultimate aim of this study was to engineer cartilage constructs *in vitro*, with both staining characteristics and mechanical properties comparable to those of native cartilage. This was attempted by first seeding bovine synoviocytes onto synthetic nonwoven PET scaffolds and culturing in a chondrogenic medium for 4 weeks. The resulting immature constructs were then subjected to cyclic compressive loading, for periods lasting 28, 56, or 84 days. The initial loading parameters were determined from the immature constructs' response to load, which indicated that cyclic strains of amplitude between 13% and 30% could produce suitable stress within the deposited matrix and residing cells, and trigger desired anabolic mechanotransductive effects. Compressive loading was achieved using a force-controlled bioreactor and silicone rings that surrounded each construct, thereby delivering the desired strain.

Within 28 days of applying compression, an increase in cell proliferation was observed, evidenced by a statistically significant increase in DNA content that was absent in nonloaded controls. The DNA content remained at an elevated level throughout the remaining culture period (total 84 days). By day 56 of applying compression, there was a substantial increase in the compressive moduli in the constructs in comparison with those observed at day 28 and in comparison with those in the nonloaded (control) constructs. This occurred in conjunction with the histological findings of intense, homogeneous Alcian blue staining and type II collagen immuno-positive staining, with low immuno-positive staining for type I collagen throughout the loaded constructs. The results indicate an effect of the applied mechanical stimulus on the resident cells within the constructs, whereby these cells have deposited extracellular matrix (ECM) that has characteristics similar to that of native cartilage. Furthermore, the modulus of the constructs reached values that were at the higher end of the range of modulus values of native cartilage.

The effects of compressive loading on tissue-engineered cartilage constructs have been previously reported and are similar in nature to the results within this study, whereby mechanical stimulus resulted in cellular differentiation and deposition of ECM, which increased the modulus of the constructs.^9,16–25^ Indeed, the response of native cartilage to mechanical stimulus is well known, and the regional variations in the compressive modulus of native cartilage have been shown to correlate with the corresponding variations in the predominating regional stress.^14,40,41^

Numerous publications have reported an increase in cell proliferation attributed to mechanical stimulation during 3D culture of chondrocytes.^42–45^ Different pathways have been implicated in such a response, involving stretch-activated sodium ion channels^44^ and integrins (specifically α_1_β_5_).^43^ Significantly, these studies suggested that proliferation caused by mechanical stimulation occurs primarily in immature chondrocytes^44^ and second when the cells are in a “receptive” phenotype (e.g., after treatment with TGF-β3).^43^ The observation that cell proliferation occurs in immature chondrocytes implies that proliferation is a natural stage in the formation of new cartilage tissue.^44^ This suggests that the synoviocytes used in this study had differentiated to an immature chondrocyte-like phenotype after the 4 weeks of chondro-induction and upon commencement of mechanical loading.

From days 0 to 28 of mechanical loading, there was a statistically significant drop in protein content for both loaded and nonloaded constructs, whereas both experimental groups displayed a significant increase in staining for cartilage matrix. This unexpected phenomenon could, therefore, be attributed to the continued action of the chondrogenic medium, which caused the noncartilage-like matrix to undergo significant turnover toward a cartilage-like matrix with an overall net drop in protein content.

The moduli of constructs produced in this study were substantially greater than any previously published values for *in vitro*-produced cartilage constructs, to our best knowledge. This is the first time in which a means is shown to form cartilage constructs with dynamic mechanical properties that compare with the *higher* range of native articular cartilage. In addition, the compressive modulus was strongly correlated with the mean intensity of Alcian blue staining in the construct sections, suggesting that it is the accumulation of suitable ECM (i.e., glycosaminoglycans) within the constructs that is associated with the mechanical properties. Although the engineered tissue is similar to native cartilage in its mechanical and matrix staining properties, we refer to it as cartilage-like because it did not exhibit the zonal variations observed in native cartilage, in particular, the presence of a calcified zone.

One aspect that may contribute to the high moduli reached in this study could lie in the scaffold used here in comparison to those used previously by others, that is, hydrogel versus nonwoven filaments. As hydrogels encapsulate cells, matrix is deposited around the periphery of the cell and, therefore, matrix initially forms in pockets with respect to the overall construct.^9,18^ Nonwoven fiber scaffolds have greater voids for the deposited matrix to fill (in this case 90.2% space void of material) and so matrix was observed throughout the scaffold. Uniformity of deposited matrix (such as that we have reported here) means that overall applied strain will directly relate to matrix strain, rather than scaffold strain. A nonwoven fiber scaffold will also enhance cell–cell communication over an encapsulating scaffold, which has been implicated in partially controlling differentiation toward a chondrogenic phenotype.^46,47^

The site-specific distribution of the matrix components might also be expected to contribute toward construct moduli, although this was not investigated in detail in this study. Huang *et al.* (2010) found that although application of compressive loading to constructs improved mechanical properties, these improvements were attributed to a change in matrix distribution and not to changes in matrix quantity.^10^ In addition, by computer simulation, Nagel and Kelly (2012) were able to predict experimental observations of constructs improving their mechanical properties after compression by structural changes in the collagen network.^48^ However, an explanation as to why the constructs in our study reached high-moduli values could be related to the nature of the mechanical regime as the constructs matured.

### Mechanism of *in vitro* engineering of cartilage-like tissue—a hypothesis

We next attempt to explain the mechanism by which the immature constructs are transformed into cartilage-like material both in staining characteristics and in mechanical properties. The following developments were observed:

(1) The thickness of the loaded constructs significantly reduced from day 0 (that is after 4 weeks of preculture) to day 28, after which it steadily increased for the remaining culture period up to day 84. The estimated applied strain followed a similar trend.
(2) The modulus of the constructs increased in step as mentioned (as shown in Table 2 and Fig. 9). Because the compressive stiffness of the constructs was gradually becoming more comparable with that of the surrounding silicone ring (and indeed eventually increased *beyond* it), the constructs would be compressed with progressively increasing load as they took a greater proportion of the load delivered by the plunger of the bioreactor.

It appears that upon mechanical loading (for 1 h per day, 5 days per week), there is initial compaction of the constructs, causing an observed decrease in thickness. Then during the periods of rest, the cells respond to mechanical loading by synthesizing and depositing cartilage-like matrix that progressively fills the voids within the scaffold, thereby increasing its stiffness and thickness. As a direct result, the stress and strain applied to the constructs by the bioreactor increase accordingly during the following periods of loading. This iterative process of increasing mechanical stimulation could be a significant contributing factor in creating cartilage-like constructs with compressive moduli that are comparable to the higher range of native articular cartilage values.

**FIG. 16.**
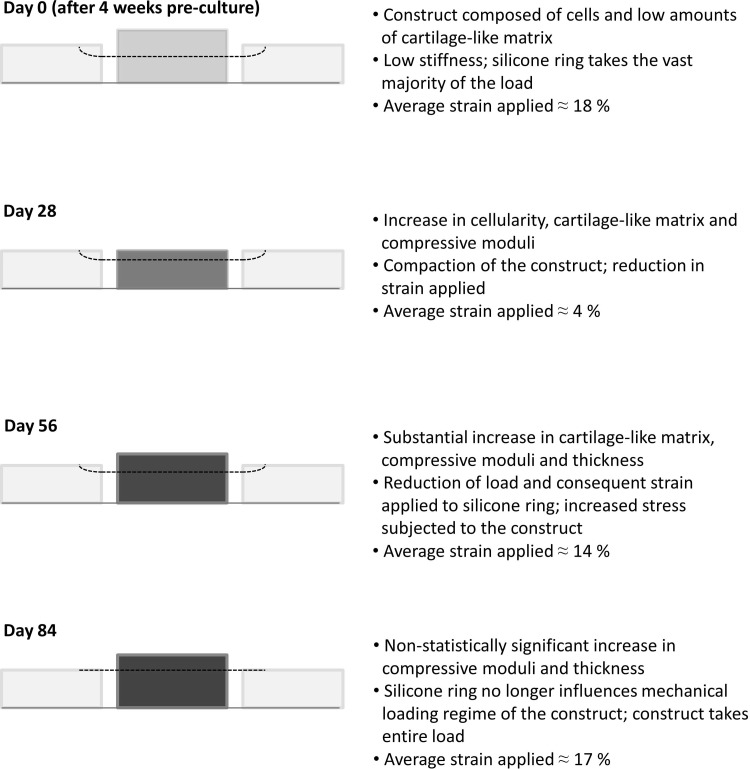
Hypothesized stages in the development of the cartilage-like constructs with the application of compressive mechanical loading. *Black dotted line* suggests the level of strain applied to the construct and silicone ring at each measured time point.

Based on our combined data, we suggest that when mechanical loading is initiated, the loading regime triggers a surge in cellular proliferation and a compaction of the constructs. After deposition of an appropriate quantity of cartilage-like matrix (primarily as a result of continuous exposure to TGF-β3), the residing cells react to the challenge of mechanical loading by upregulating the cartilage-like ECM net synthesis and secretion in an attempt to “resist” the applied load. As the cells continue to lay down cartilage-like matrix, the ECM will spread (and would be expected to transmit) the load away from the cells, reducing their stimulation. However, because of the bioreactor setup, as the constructs' stiffness and thickness increase, the stress and strain applied onto the constructs also increase, thereby maintaining cellular mechanical stimulation, inducing continued anabolic effects. This would continue until the majority of the load applied from the bioreactor is taken by the constructs and not by the surrounding silicone ring. At this point, the mechanical loading to the constructs will have reached a plateau and will act to simply maintain tissue composition akin to the homeostasis of native articular cartilage.^14^ This hypothesis is summarized in Figure 16.

Using an increasing magnitude of applied load over time to promote matrix deposition has not been reported previously in the literature. However, a study by Khoshgoftar *et al.* (2013), using finite element analysis, suggested that an increase in applied load would be required to maintain cellular loading as pericellular matrix increased,^49^ supporting the experimental evidence within this study.

The initial strategy of selecting a loading regime based on the mechanical properties of precultured constructs was seemingly an important step in creating the initial baseline mechanical culture conditions, by furthering the differentiation of the residing cells as evidenced by triggered proliferation. However, the data suggest that the presence of a self-increasing loading regime (that is driven by the maturation of the developing constructs themselves) appears to have been a key feature, and most significant finding of this study, to produce cartilage-like constructs with high moduli.

The rate at which each construct progressed along the overall growth trajectory appeared to be individual. Despite starting with the same conditions (scaffold, seeding density, and chondrogenic medium), variations in construct thickness and stiffness were first measured after 4 weeks in the chondrogenic culture and there was clear variation at later time points, with or without the presence of mechanical loading. A self-adapting mechanism proposed here, delivered by a multichannel bioreactor (with one construct per loading station), would be advantageous to the seemingly inherent variability of tissue-engineered, biological samples. The majority of bioreactors, to date, have used one loading mechanism to apply load to multiple constructs^9,17,20,22–24,50,51^ and, therefore, may not deliver optimum stimulation to each individual sample during its growth. The choice of scaffold (nonwoven filamentous, rather than hydrogel), the use of a multichannel bioreactor, and the self-regulating mechanical loading regime appear to be the significant aspects of this study to form constructs with high stiffness and cartilage-like appearance over previous studies.

It remains unknown to what exact construct properties are required to elicit repair. In a recent study by Fisher *et al.* (2014),^52^ they suggest that the most suitable time to implant a construct should be based not only on the mechanical and biochemical properties at a given time point, but also on the time in which the highest rate of change is occurring (with regard to increase in cartilage components and mechanical properties). This was based on a static *in vitro* integration experiment. This would suggest that implantation should occur when the mechanical properties are sufficient to survive and transmit load within the joint, and when the residing cells are at their most active. This would appear to expedite the formation of new cell–cell and cell–matrix interactions plus increased matrix turnover, which improves the rate and chances of integration to occur. Fisher *et al.* are currently investigating this strategy with *in vivo* experiments and this strategy could be applied to the constructs in this study.

Although high-compressive moduli values were achieved in this study, there remains scope for further optimization to improve the rate of quality matrix deposition and further understanding of the mechanical cues (such as tension and shear triggered by uniaxial compression), to which the residing cells and matrix could be subjected to. Nevertheless, we have illustrated that a self-regulating system, which increases the applied stress and strain as the constructs' stiffness and thickness increase, has the potential to produce constructs with moduli values equal to those of native cartilage, raising the possibility of improved clinical outcomes when such constructs are used as a direct tissue replacement. Such a construct could be produced from autologous cells, or alternatively decellularized to create an “off the shelf” replacement tissue.

## Conclusions

In an attempt to sufficiently load the residing matrix and cells of precultured constructs to accelerate the deposition of cartilage-like matrix and production of high modulus constructs, an approach of measuring the constructs' mechanical response to load to select an initial loading regime was undertaken. The applied regime initially caused a surge in cell proliferation and continued cartilage-like matrix deposition from static chondrogenic culture. We hypothesize that what followed was a self-regulating mechanical regime that increased the applied stress and strain in conjunction with the maturing constructs' stiffness and thickness, which was generated by the bioreactor setup. These two processes initialized and maintained significant mechanical cell stimulation to form cartilage-like constructs with both histological appearance and high compressive moduli comparable to those of native cartilage, far greater than nonloaded controls and other cartilage constructs described in the literature. Hence, the constructs could be mechanically functional as a direct tissue replacement for cartilage repair.
